# CSF pulsations measured in Parkinson’s disease patients using EPI-based fMRI data

**DOI:** 10.3389/fnagi.2024.1369522

**Published:** 2024-04-26

**Authors:** Jun-Hee Kim, Suhong Kim, Jae-Geun Im, Seok Jong Chung, Phil Hyu Lee, Yong Jeong, Sung-Hong Park

**Affiliations:** ^1^Department of Bio and Brain Engineering, Korea Advanced Institute of Science and Technology, Daejeon, Republic of Korea; ^2^Department of Radiology, Yeungnam University College of Medicine, Daegu, Republic of Korea; ^3^Yonsei University College of Medicine, Seoul, Republic of Korea; ^4^Yongin Severance Hospital, Yongin, Republic of Korea

**Keywords:** CSF pulsation, Parkinson’s disease, dementia, functional MRI, brain clearance

## Abstract

**Introduction:**

Cerebrospinal fluid (CSF) flow is involved in brain waste clearance and may be impaired in neurodegenerative diseases such as Parkinson’s disease. This study aims to investigate the relationship between the CSF pulsation and the development of dementia in Parkinson’s disease (PD) patients using EPI-based fMRI.

**Methods:**

We measured CSF pulsation in the 4th ventricle of 17 healthy controls and 35 PD patients using a novel CSF pulsation index termed “CSFpulse” based on echo-planar imaging (EPI)-based fMRI. The PD patients were classified into a PD with dementia high-risk group (PDD-H, *n* = 19) and a low risk group (PDD-L, *n* = 16), depending on their development of dementia within 5 years after initial brain imaging. The size of the 4th ventricle was measured using intensity-based thresholding.

**Results:**

We found that CSF pulsation was significantly higher in PD patients than in healthy controls, and that PD patients with high risk of dementia (PDD-H) had the highest CSF pulsation. We also observed an enlargement of the 4th ventricle in PD patients compared to healthy controls.

**Conclusion:**

Our results suggest that CSF pulsation may be a potential biomarker for PD progression and cognitive decline, and that EPI-based fMRI can be a useful tool for studying CSF flow and brain function in PD.

## Introduction

Recently, cerebrospinal fluid (CSF) flow has been highlighted for its function of brain waste clearance (Louveau et al., 2017). CSF flow is involved in the glymphatic system, which outflows waste products from parenchyma through the exchange with interstitial fluid (ISF) and CSF (Kaur et al., 2021). Furthermore, CSF flow also facilitates the transfer of waste from the CSF to the meningeal lymphatic vessels (mLVs), eventually leading to the cervical lymphatic nodes (Kaur et al., 2021). This also highlights the importance of CSF flow in the efficient removal of waste from the brain.

Previous studies reported that impairment in brain clearance steps is associated with neurodegenerative diseases, cognitive deficit, and central nervous disorders (Da Mesquita et al., 2018). There are representative neurodegenerative diseases thought to be associated with defect in CSF flow such as Alzheimer’s disease and Parkinson’s disease. For instance, in Alzheimer’s disease, brain amyloid-beta (Aβ) deposition is believed to be a consequence of impaired Aβ clearance. CSF clearance, a significant aspect of brain glymphatic system and mLVs clearance, has been shown to be abnormal in murine models of Alzheimer’s disease (Li et al., 2022). Similar to Alzheimer’s disease, one of the major causes identified in Parkinson’s disease is the phenomenon of “prion-like protein propagation.” In Parkinson’s disease, the accumulation of the alpha-synuclein protein is associated with the progression of the disease (Buccellato et al., 2022). It is hypothesized that impairment in the clearance of alpha-synuclein through the brain clearance system could be related to Parkinson’s disease (Buccellato et al., 2022).

There is insufficient research that approaches Parkinson’s disease from the perspective of brain clearance in relation to CSF flow. Based on the previous studies, correlation between global-BOLD and CSF inflow was significantly low in both Alzheimer’s disease and Parkinson’s disease with dementia (Han et al., 2021a,b). In addition, there is a study that showed the results of distinguishing Parkinson’s disease patients from progressive supranuclear palsy patients by using CSF flow measurements through phase contrast MRI (Fukui et al., 2015). However, the direct association between ventricular CSF pulsation and Parkinson’s disease has not been sufficiently investigated. Numerous preceding studies published thus far have extensively utilized EPI-based fMRI in researching cognitive brain function and brain networks in Parkinson’s disease (Baggio et al., 2014; Aarsland et al., 2021). In this study, we aimed to demonstrate the relationship between CSF pulsation and cognitive deficit development in Parkinson’s disease (PD) patients using a novel CSF pulsation measurement technique based on conventional EPI-based fMRI (Kim et al., 2022, 2024).

## Materials and methods

All the experiments were performed on a 3T whole-body scanner (Phillips). This study was approved by local Institutional Review Board (Chung et al., 2022). We used fMRI data from 17 healthy controls (HC) and 35 patients with PD. The PD patients were classified into a PD with dementia high-risk group (PDD-H, *n* = 19) and a low risk group (PDD-L, *n* = 16), depending on their development of dementia within 5 years after initial brain imaging. In other words, we conducted an fMRI scan at the very beginning and the PD group classification was carried out after 5 years. The diagnosis of PDD was made according to the clinical diagnostic criteria proposed by the Movement Disorder Society Task Force (Emre et al., 2007; Yoo et al., 2019). The demographics and mini-mental state examination (MMSE) scores, disease duration, and Unified Parkinson’s Disease Rating Scale (UPDRS) scores are shown in Table 1. The Parkinson’s disease is affected by and often assessed through various clinical characteristics. Thus, we conducted statistical tests based on demographic data, including age, gender, and education.

**TABLE 1:** The clinical demographic data of dataset and comparison of clinical demographic data between the groups.

				Difference between groups (*p*-value)			
	Healthy control (*n* = 17)	PDD-L (*n* = 16)	PDD-H (*n* = 19)	HC PDD	HC PDD-L	HC PDD-H	PDD-H PDD-L
Age (years)	72.3 ± 7.4	69.2 ± 9.3	75.3 ± 7.7	0.93	0.31	0.25	0.05
Gender^A^ (M/F)	10 / 7	11 / 5	13 / 6	0.53	0.72	0.5	1
Education (years)	10.1 ± 3.4	8.3 ± 5.4	7.9 ± 5.5	0.17	0.27	0.17	0.85
MMSE*	–	26.1 ± 4.0	21.7 ± 4.0	–	–	–	0.003
Duration (years)	–	11.8 ± 8.5	20.7 ± 16.1	–	–	–	0.06
UPDRS	–	29.4 ± 6.5 (*n* = 11)	26.5 ± 8.1 (*n* = 15)	–	–	–	0.36

*p < 0.05; there was a significant difference between the groups, ^A^Fisher’s exact test.

For resting-state fMRI, 2D multi-slice EPI images were acquired with following parameters: repetition time/echo time/flip angle = 2,000 msec/30 msec/90°, resolution = 2.75 mm^2^ × 2.75 mm^2^, slice thickness = 4 mm, matrix size = 80 × 80, slice order = ascending interleaved, number of slices = 31. Total 160 measurements were performed for the resting-state fMRI with the whole brain coverage. All the EPI datasets were preprocessed using FSL FEAT (Woolrich et al., 2001, 2004), including temporal high pass filter (0.01 Hz), motion correction (MCFLIRT) and slice-timing correction (Kim et al., 2022).

**FIGURE 1:**
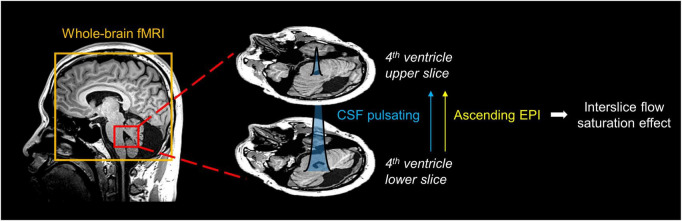
Schematic diagram for measurement of CSF pulsation from EPI imaging. The interslice pulsated CSF signal in 4th ventricle was selected. The pulsation direction of inflowing CSF matched the ascending EPI imaging order, thus the interslice flow saturation effect could be used to measure CSF pulsation.

To measure CSF pulsation from EPI-based fMRI data, we applied the CSFpulse technique, which utilizes the interslice flow saturation effect (Park and Duong, 2011; Park et al., 2012; Kim et al., 2022, 2023). Simulation of EPI signal and CSF pulsation modeling (Kim et al., 2022) enabled the measurement of CSF pulsation, considering CSF signals and interslice saturation effects within EPI inner slices across multiple measurements based on the matrix driven Bloch equation (Benoit-Cattin et al., 2005). In the previous study, the proposed CSFpulse was highly correlated with stroke volume measured with phase contrast MRI in the aqueduct, which reflects ventricular CSF pulsation (Kim et al., 2022). CSF signals from the two nearby 4th ventricle slices were used to calculate the interslice CSF pulsation (Figure 1). The quantitative metric of CSF pulsation (CSFpulse) was calculated as below to represent the interslice pulsed CSF volume.


(1) $$\text{CSFpulse}\,\left(\text{n}\right)=\left(\frac{1}{\alpha -1}\right)\times \left(\frac{S_{i}\,\left(n\right)}{S_{i-1}\,\left(n\right)}-1\right)\times R\,O\,I\,v\,o\,l\,u\,m\,e$$

where S_i_(*n*) indicates the ith slice CSF signal intensity in the nth measurement, α indicates the ratio between the pulsating CSF signal and non-pulsated steady state CSF signal, and *ROIvolume* represents the volume of each MRI voxel (Supplementary Figure 1; Kim et al., 2022). For measurements with CSFpulse(n) < 0, then CSFpulse(n) was set to 0. It should be noted that based on the previous study, averaging CSFpulse more than 7 measurements showed a statistically significant correlation with the phase contrast stroke volume, and the correlation coefficient significantly increased further after averaging more than 80 sessions of CSFpulse (Kim et al., 2022).

Based on the default settings of Philips, the interleaved ascending order skips 1 plus the square root of the number of slices. This means that EPI slices (total 31 slices) were acquired in the order of 1, 7, 13, 19, 25, 31, 2, 8, 14, 20, 26, 3, 9, 15, 21, 27, and so on up to 6, 12, 18, 24, 30. Consequently, we set the target slice pair of the 4th ventricle by the nearby slices (for example, Slices 8 and 9). All the 4th ventricle target slices were located between 6∼11. In this scenario, the RF interval time between two target slices (interTR) was 322 ms in most cases, and 387 ms only when the lower slice from the paired slices was slice number 6 or 7. The CSFpulse results of this study were processed based on a new simulation with interTR = 322 ms or 387 ms (interTR = 322 ms case for Supplementary Figure 1). Based on this scheme, the captured velocity range of CSF would be 0 − 2.48 cm/s (8 mm/322 ms) or 0 − 2.06 cm/s (8 mm/387 ms).

The target 4th ventricle slices were selected manually and the region-of-interest (ROI) of 4th ventricle CSF was mapped automatically based on the intensity thresholding. Then, CSFpulse was calculated from 155 EPI measurements (excluding the first 5 measurements), where each CSFpulse indicated single pulsation amount during the scan. The mean and the z-score of the dynamic CSFpulse were calculated for each subject to represent the strength and variability of CSFpulse during the resting state. The number of CSF ROI voxels was counted for each subject. The cross-sectional area of the CSF ROI in the 4th ventricle was compared across subjects based on the number of CSF ROI voxels.

All the statistical tests were conducted using SPSS (version 25; IBM Corp.) and MATLAB R2020a (Mathworks). To compare the difference in CSFpulse and the number of CSF ROI voxels between HC and PDD groups, two-sample *t*-test was conducted for statistical evaluation. The gender distribution difference was assessed for the groups with the Fisher exact test.

## Results

The mean and standard deviation of the demographic data is demonstrated in Table 1. Due to lack of UPDRS data, we could use 11 and 15 data of PDD-L and PDD-H, respectively. In our dataset, only MMSE between PDD-L and PDD-H showed significant difference (Table 1). There were differences between PDD-L and PDD-H in disease duration (PDD-L: 11.8 ± 8.5 years, PDD-H: 20.7 ± 16.1 years) and age (PDD-L: 69.2 ± 9.3 years, PDD-H: 75.3 ± 7.7 years), although these differences did not reach statistical significance (Table 1). However, none of the metrics from the groups showed a significant correlation with CSFpulse (Supplementary Table 1).

**FIGURE 2:**
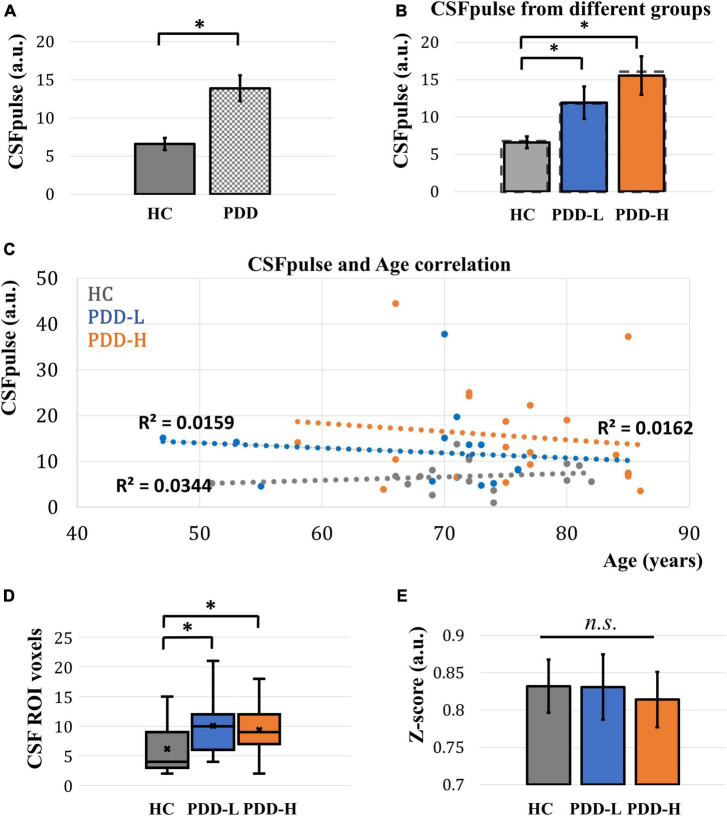
Acquired CSFpulse from different groups and its comparison. **(A)** Comparison of CSFpulse between HC group and PD group. **(B)** Comparison of CSFpulse between HC, PDD-L and PDD-H groups. Dashed line represents for the age controlled CSFpulse. **(C)** The scatter plot of correlation between CSFpulse and age across the all dataset. **(D)** Comparison of number of CSF ROI voxels in 4th ventricle target slice. **(E)** Comparison of averaged absolute z-score from CSFpulse between HC, PDD-L and PDD-H groups. Each bar graph represents the mean CSFpulse and its standard error. HC: healthy control group, PDD-L and PDD-H: Parkinson’s disease with dementia low-risk and high-risk groups. *p < 0.05; there was a significant difference between the groups.

In the demographic data, age, gender, and education were control factors across the groups, and there was a difference in age between the PDD-L and PDD-H groups (Table 1). Thus, we utilized a two-sample *t*-test to analyze differences in both the normal CSFpulse and age-controlled CSFpulse between the groups. Both before and after adjusting the age covariance from CSFpulse, CSFpulse values from different groups showed significant difference (*p* < 0.05; two-way ANOVA and Bonferroni) (Figure 2A and Table 2). In case of CSFpulse, PDD-H and PDD-L were significantly higher than those of the HC (*p* < 0.05; two-sample *t*-test) (Figure 2B and Table 2). Difference in CSFpulse between PDD-L and PDD-H was not statistically significant (*p* = 0.292; two-sampled *t*-test) (Figure 2B and Table 2). In case of CSFpulse with age adjustment, PDD-H CSFpulse was significantly higher than the CSFpulse of HC group (*p* < 0.05; bootstrap for pairwise comparisons) (Figures 2B, C and Table 2). PDD-L CSFpulse also showed high pulsatility than HC group (*p* = 0.065; 95% confidence level −0.297∼−0.19; bootstrap for pairwise comparisons) (Figures 2B, C and Table 2).

**TABLE 2:** The quantitative values of CSFpulse, age controlled CSFpulse, number of CSF ROI voxels and average absolute z-score in healthy control, PDD-L and PDD-H.

	Healthy control (*n* = 17)	PDD-L (*n* = 16)	PDD-H (*n* = 19)
CSFpulse* (a.u.)	6.6 ± 0.86	11.9 ± 2.2	15.6 ± 2.6
Age controlled CSFpulse* (a.u.)	6.7 ± 2.1	11.6 ± 2.2	15.8 ± 2.0
Number of ROI voxels*	6.23.9	10.1 ± 4.3	9.4 ± 3.7
Average Z-score of CSFpulse (a.u.)	0.83 ± 0.036	0.83 ± 0.044	0.81 ± 0.037

*p < 0.05; there was a significant difference between the groups.

There was no difference in number of CSF ROI voxels between PDD-L group and PDD-H group (*p* = 0.62; two-sampled *t*-test) (Figure 2D and Table 2). However, both PDD-L and PDD-H showed larger CSF ROIs in the 4th ventricle compared to those of the HC group (*p* < 0.05; two-sampled *t*-test) (Figure 2D and Table 2).

During the dynamic fMRI acquisition, CSF pulsation amplitude can be changed. This unique characteristic, typically observed during sleep, is known to be an indicator of alterations in CSF circulation and clearance (Fultz et al., 2019). To evaluate this pulsation variability, we checked the absolute z-score of the dynamic CSFpulse data. Although the absolute z-score of PDD-H was slightly lower than the others, there was no significant difference in the absolute z-scores across the groups (two-sampled *t*-test for each pair of groups) (Figure 2E and Table 2).

## Discussion

In this study, we investigated the CSF pulsation of Parkinson’s disease patients using the CSFpulse technique based on the EPI-based fMRI. As a result, CSFpulse increased in PD group than HC group, both PDD-L and PDD-H showed a significant difference in CSF pulsation compared to HC group, and PD patients with high risk of dementia showed the highest CSFpulse (Figure 2B). The measured CSFpulse is determined by two factors: the ratio of saturated CSF signals in target CSF ROIs and the volume of ROI (Eq. 1). The ROI volume of 4th ventricle in the target slice (S_i_) demonstrated an enlargement in PD patients compared to the HC group (Figure 2D). In a separate study, we also measured the 4th ventricle volume based on the 3D-T1 structural images and examined the correlation between the two measured 4th ventricle volumes (Supplementary Figure 2). We discovered a high correlation between the 4th ventricle CSF ROI size from the EPI-fMRI data and the 4th ventricle volume from the 3D-T1 structural images, suggesting that the 4th ventricle CSF ROI size adequately represents the 4th ventricle volume (Supplementary Figure 3).

Previous studies have indicated that the size of the 4th ventricle in PD with mild cognitive impairment was significantly larger than that of HC, showing high correlation with memory performance in these patients (Dalaker et al., 2011). Additionally, lateral ventricular enlargement has shown potential as a structural biomarker for PD (Apostolova et al., 2012). Moreover, the maximum CSF velocity of aqueduct measured using phase contrast technique was significantly higher in the PD patient group compared to the non-PD patient group (Fukui et al., 2015). Thus, the increment of CSF pulsation in PD than HC in this study could be significantly attributed to ventricular enlargement in the PD patient group. Between PD dementia low-risk and high-risk groups, there was no difference in ROI volume (Figure 2D). Furthermore, in this study, our dataset showed no significant correlation between age and 4th ventricle CSF ROI size (All subjects; *n* = 52; Pearson correlation *p*-value = 0.53), and no significant correlation between MMSE and 4th ventricle CSF ROI size (PD patients; *n* = 35; Pearson correlation *p*-value = 0.7) (Supplementary Figure 4). However, the CSFpulse was slightly higher in the PDD-H group than the PDD-L group (Figure 2B), although the difference could not reach the statistical significance. This suggests that the other factor, the ratio of saturated CSF signals (CSF pulsation amount), also plays a significant role in determining the CSFpulse difference among the groups, especially in PDD-H. Another study demonstrated that total arterial cerebral blood flow (tCBF) and the calculated pulsatility index from aqueduct phase-contrast MRI were higher in mild cognitive impairment patients (El Sankari et al., 2011), aligning somehow with our observations. However, it should be noted that the MRI scan in this study was conducted 5 years earlier than the assessment point for categorizing individuals into dementia low or high-risk groups, and thus the correlation could have weakened or changed during the time gap.

The CSFpulse measurement occurs when the CSF flows within a cut-off velocity range (yielding a positive CSFpulse). Therefore, a negative CSFpulse could occur when the CSF flow velocity at that measurement falls outside the cut-off velocity range (0∼2.06 or 2.48 cm/s), or when the CSF flows in the opposite direction (descending) at the moment of measurement. Thus, when examining the positive CSF pulse ratio, it can be interpreted that subjects with a high positive ratio experienced more CSF pulsations within the cut-off velocity range, whereas the subjects with a low positive ratio may not necessarily have faster mean CSF velocity but rather may have relatively frequent occurrences of larger pulsations that exceed cut-off velocity range or of opposite-directional pulsations resulting in negative velocities. We compared the positive CSFpulse ratio among HC, PDD-L, and PDD-H using 155 measurements. While HC and PDD-L exhibited similar ratios, PDD-H showed a higher positive ratio (Supplementary Figure 5). Although the difference in positive ratio between PDD-L and PDD-H did not reach statistical significance, it demonstrated a difference approaching statistical significance (paired *t*-test; *p* = 0.065). This result is somehow consistent with the lower pulsation variability of PDD-H shown in Figure 2E. These results suggest that CSF pulsation in PDD-H could be more biased in the ascending direction or PDD-H patients may experience less frequent occurrences of large pulsations intermittently compared to PDD-L and HC. This periodic large CSF pulsation phenomenon was observed in a previous fast fMRI study, which reflects the important brain clearance through CSF pulsation associated with sleep (Fultz et al., 2019).

Furthermore, CSF pulsation might be actively increased as a result of greater cerebral waste clearance deposits in Parkinson’s disease patients. In addition, the progression of PD and subsequent dementia development might be affected not only by CSF pulsation amplitude, but also by factors such as the meningeal lymphatic function (often impaired with aging), the efficiency of the glymphatic system responsible for clearance, or the coupling between CSF inflow and global brain activity (Ahn et al., 2019; Han et al., 2021a,b). However, it is important to note limitations in interpreting the results of this study, such as the limited number of subjects and the inability to accurately or systematically assess factors such as smoking habits, alcohol consumption, or physical activity.

In summary, our study demonstrated a correlation between CSF pulsation observed in fMRI data and the progression of Parkinson’s disease as well as the subsequent dementia development after Parkinson’s disease onset. This correlation suggests potential application to studying brain function in Parkinson’s disease and its associated dementia, particularly in understanding brain clearance mechanisms related to CSF pulsation.

## Data availability statement

The data analyzed in this study is subject to the following licenses/restrictions: MRI data and the scripts for this research will be made available upon reasonable request to the corresponding authors after permission by institutional review board. The processing codes for CSFpulse that support the findings of this study are openly available in GitHub at github.com/KAIST-MRI-Lab/JunheeKim. Requests to access these datasets should be directed to S-HP, sunghongpark@kaist.ac.kr.

## Ethics statement

The studies involving humans were approved by the Yonsei University Severance Hospital Institutional Review Board (4-2020-0822). The studies were conducted in accordance with the local legislation and institutional requirements. The Ethics Committee/Institutional Review Board waived the requirement of written informed consent for participation from the participants or the participants’ legal guardians/next of kin because of the retrospective nature of the study.

## Author contributions

J-HK: Conceptualization, Formal analysis, Investigation, Methodology, Visualization, Writing – original draft, Writing – review & editing. SK: Conceptualization, Data curation, Formal analysis, Investigation, Methodology, Writing – review & editing. J-GI: Conceptualization, Formal analysis, Investigation, Methodology, Writing – review & editing. SC: Data curation, Writing – review & editing. PL: Data curation, Writing – review & editing. YJ: Conceptualization, Methodology, Supervision, Writing – review & editing. S-HP: Conceptualization, Funding acquisition, Methodology, Supervision, Writing – original draft, Writing – review & editing.
