# Supplementary material for: CSF pulsations measured in Parkinson’s disease patients using EPI-based fMRI data
Source: Front Aging Neurosci. 2024 Apr 26;16:1369522. doi: 10.3389/fnagi.2024.1369522 (PMC11082335; doi:10.3389/fnagi.2024.1369522)
Supplement: Supplementary file 1 [file Data_Sheet_1.DOCX]

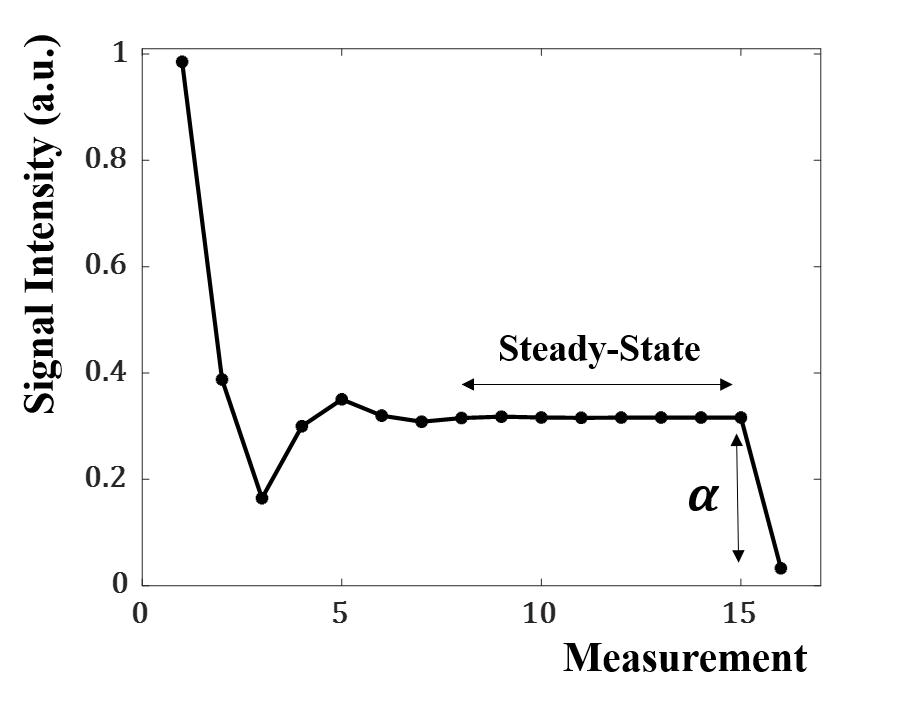


**Supplementary Figure 1.** Simulated CSF signal intensity in an EPI image with given TR/TE and CSF T1/T2. The α indicates the ratio between steady state CSF signal and the CSF signal after pulsation.

Supplementary Table 1. The correlation between CSFpulse and clinical demographic data across the groups.

| **Correlation with CSFpulse**  **(p-value)** | **Healthy Control** | **PDD** | **PDD-L** | **PDD-H** | **All subjects** |
| --- | --- | --- | --- | --- | --- |
| **Age (yrs.)** | 0.56 | 0.5 | 0.25 | 0.54 | 0.9 |
| **Education (yrs.)** | 0.73 | 0.95 | 0.38 | 0.61 | 0.7 |
| **MMSE** | - | 0.91 | 0.06 | 0.38 | - |
| **Duration (yrs.)** | - | 0.18 | 0.68 | 0.51 | - |
| **UPDRS** | - | 0.8 | 0.31 | 0.38 | - |


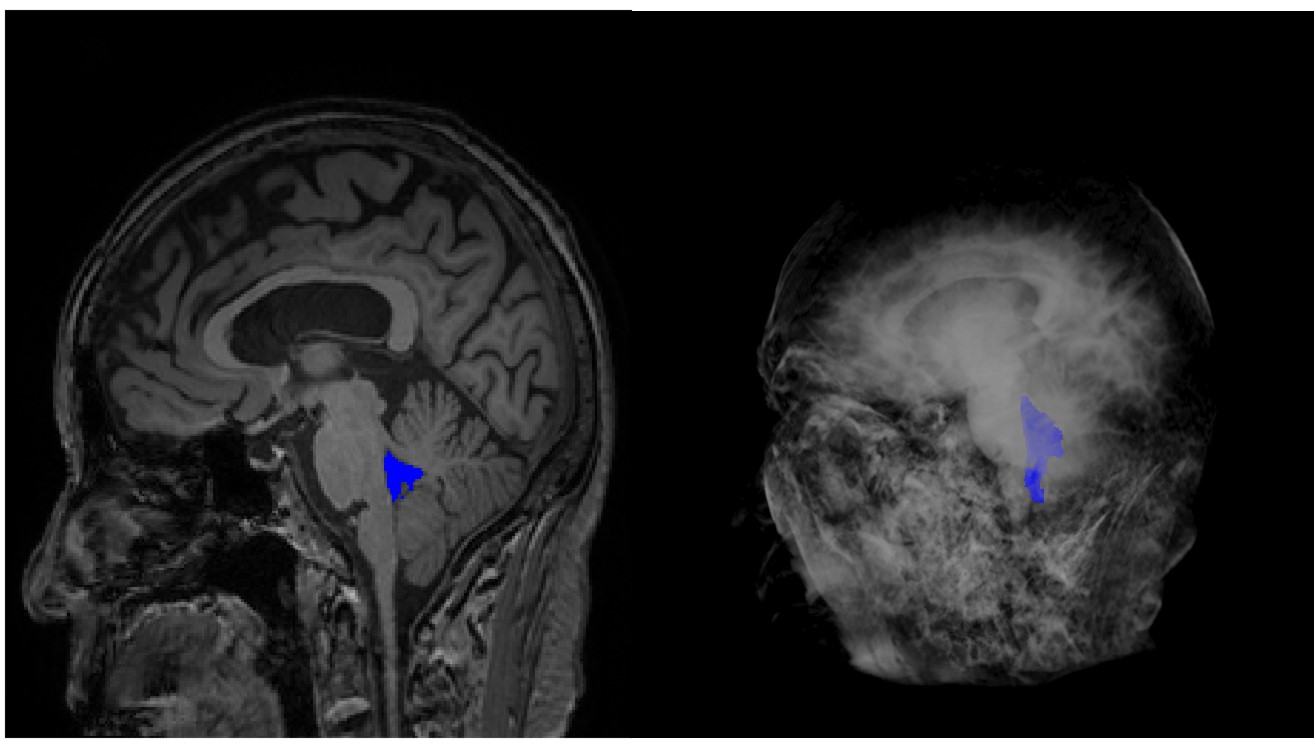


**Supplementary Figure 2.** The 4th ventricle segmentation to measure 4^th^ ventricular volume. Blue volume was segmented as a 4^th^ ventricle by FreeSurfer.


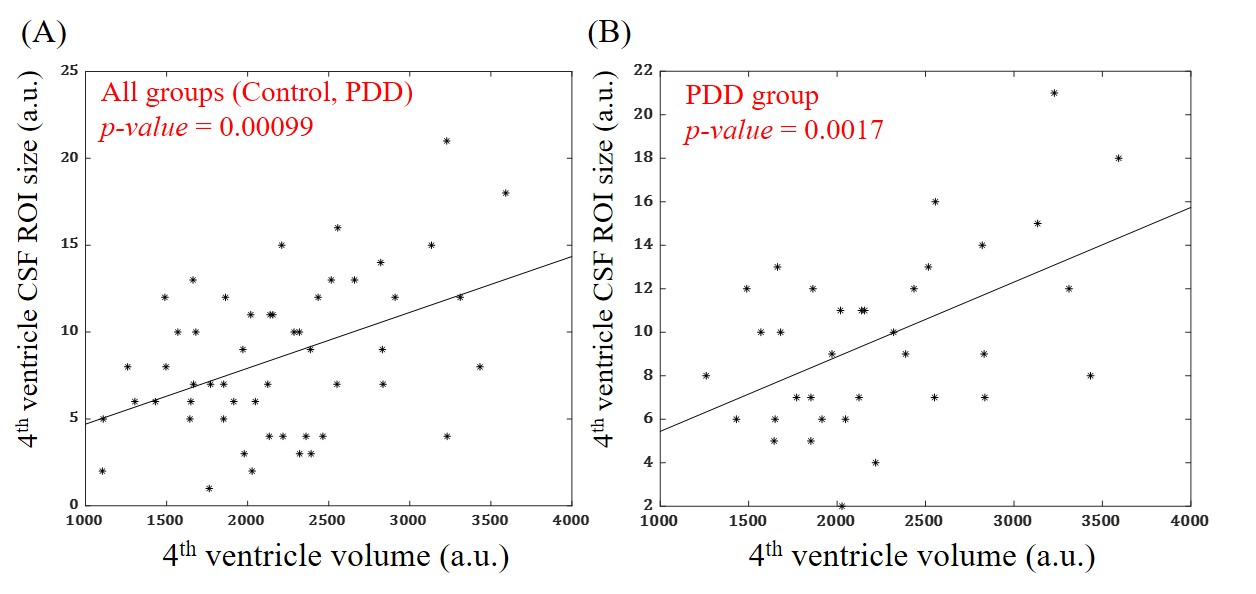


**Supplementary Figure 3.** The comparison between 4^th^ ventricle volume and CSF ROI size of 4^th^ ventricle slice. **(A)** Pearson correlation from all subject’s dataset (n=52). **(B)** Pearson correlation from PD patient group (n=35). The 4th ventricle volume and CSF ROI size of 4th ventricle slice were from the 3D T1-weighted structural images and the EPI-fMRI, respectively.


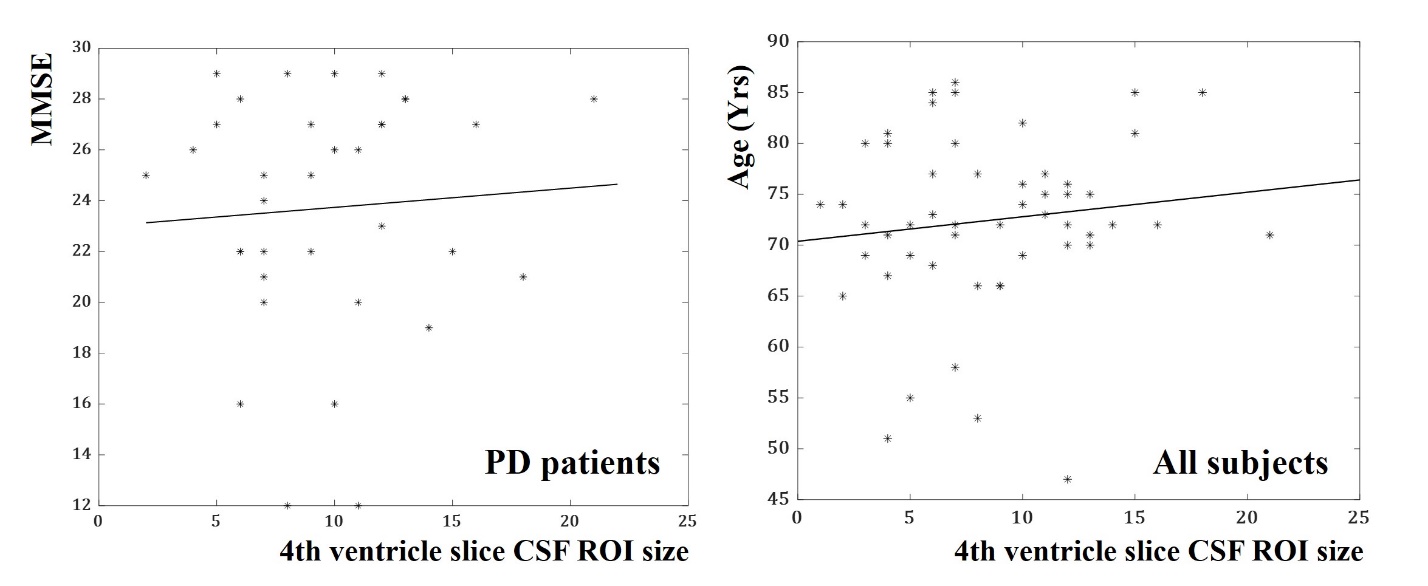


**Supplementary Figure 4.** The scatter plot of correlation between 4^th^ ventricle slice CSF ROI size and age or MMSE across the dataset.


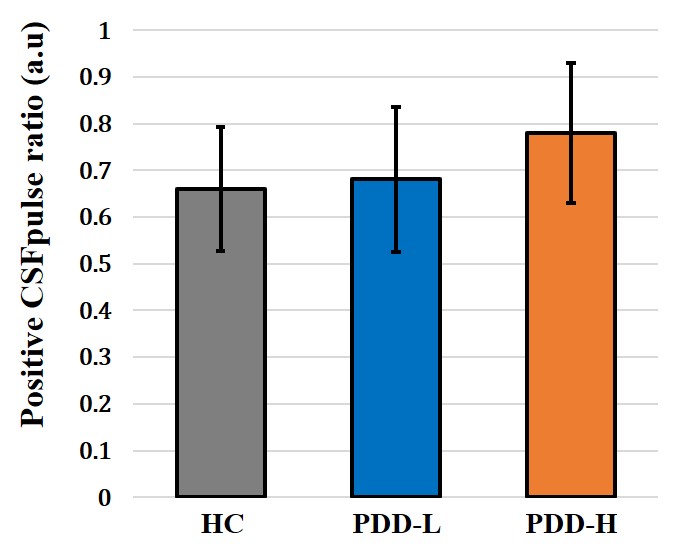


**Supplementary Figure 5.** The positive CSFpulse ratio comparison between the groups. Each bar graph represents the mean positive CSFpulse ratio and its standard deviation.
